# The level of haemoglobin in anaemic cancer patients correlates positively with quality of life

**DOI:** 10.1038/sj.bjc.6600247

**Published:** 2002-04-22

**Authors:** M Lind, C Vernon, D Cruickshank, P Wilkinson, T Littlewood, N Stuart, C Jenkinson, P Grey-Amante, H Doll, D Wild

**Affiliations:** Princess Royal Hospital, Salthouse Road, Hull HU8 9HE, UK; Oncology Departrment, Hammersmith Hospital, Du Cane Road, London W12 OH3, UK; Gynaecological Oncology Centre, South Cleveland Hospital, Middlesborough TS4 3BW, UK; Christies Hospital NHS Trust, Wilmslow Road, Withington, Manchester M20 4BX, UK; Department of Haematology, John Radcliffe Hospital, Headington, Oxford OX3 9DU, UK; Consultant Medical Oncologist, Ysbyty Gwynedd, Bangor, Gwynedd LL57 2PW, UK; Health Services Research Unit, Institute of Health Sciences, University of Oxford, Old Road, Headington, Oxford OX3 7LF, UK; Oxford Outcomes, Bury Knowle Coach House, Old High Street, Headington, Oxford OX3 9HY, UK

**Keywords:** haemoglobin, quality-of-life, anaemia, cancer

## Abstract

The aim of this study was to assess the relationship between haemoglobin level and quality-of-life in anaemic cancer patients. Patients, diagnosed with one of four cancers, were recruited if their haemoglobin level was <12 g dl^−1^ (female) or <13 g dl^−1^ (male). The condition-specific Functional Assessment of Cancer Therapy – Anaemia and the generic SF-36 were used to assess quality-of-life. Thirty-six per cent of the 179 recruited patients had breast cancer, 28% ovarian cancer, 25% lung cancer, and 11% multiple myeloma. Their mean (s.d.) haemoglobin level was 10.66 (1.04) g dl^−1^. Partial correlations controlling for the potentially confounding effects of age, gender, and time since diagnosis found significant positive relationships between haemoglobin and all domains of the Functional Assessment of Cancer Therapy – Anaemia, and with all but two of the SF-36 domains. On linear regression controlling for the same factors, each unit haemoglobin rise equalled an average 8.19 Functional Assessment of Cancer Therapy – Anaemia, and an average 6.88 Functional Assessment of Cancer Therapy–Fatigue, increase. Haemoglobin accounted for a similar amount of variability (8%) in SF-36 scores. In conclusion, quality-of-life has been found to be significantly positively related to haemoglobin level in anaemic cancer patients. This suggests that normalisation of haemoglobin in cancer patients is likely to increase their quality-of-life. The greater sensitivity of the condition-specific Functional Assessment of Cancer Therapy – Anaemia compared with the generic SF-36 suggests that the Functional Assessment of Cancer Therapy – Anaemia can be used alone to assess quality-of life in this patient group.

*British Journal of Cancer* (2002) **86**, 1243–1249. DOI: 10.1038/sj/bjc/6600247 www.bjcancer.com

© 2002 Cancer Research UK

## 

Anaemia is defined as ‘a deficiency of either red blood cells (RBCs) or lack of haemoglobin, which leads to a reduction in the oxygen-carrying capacity of blood’ (Yellen *et al*, 1997). This deficiency may result from shortened red blood cell survival, deranged iron metabolism, or relative erythropoietin deficiency (Vijayakamar *et al*, 1993). Anaemia is a common condition in patients with malignancy. Up to 30% of patients with solid tumours are reported to have anaemia, with anaemia being particularly common among those with more advanced disease (Malik *et al*, 1998). While anaemia is associated with most chronic conditions, the anaemia of malignancy may be a consequence of both myelosupression of stem cells by tumour cell products, such as tumour necrosis factor, and cytotoxic therapy. Anaemia is associated with symptoms of fatigue, lethargy, dyspnoea, loss of appetite and inability to concentrate. Fatigue, a multifactorial symptom, is the most frequently reported symptom in cancer patients (Winningham *et al*, 1994). However, despite the high prevalence of cancer-related fatigue, and its improvement with increase in haemoglobin level (e.g. Glaspy *et al*, 1997), its specific aetiology is not well understood (Cella, 1997b). Related to the reported fatigue are effects on the patient's subjective sense of well-being in that the chores of everyday life become a burden, exercise tolerance is decreased and social activity is curtailed due to lack of energy (Leitgeb *et al*, 1994). Thus the symptoms of anaemia involve both physical and emotional problems with consequent deterioration in health related quality-of-life.

Health-related quality of life (HRQoL) is a broad, subjective, and multidimensional concept that encompasses physical health and symptoms, functional status and activities of daily life, mental well-being and social health, including social role functioning. This distinguishes between quality of life (QoL) in its more general sense (an ill-defined term, with most definitions emphasising components of happiness and satisfaction with life) and QoL as appropriate for the requirements of clinical medicine and clinical trials (Fayers and Machin, 2000). The broadness of the definition of HRQoL means that multidimensional scales are needed in its measurement (Spilker, 1990; Bowling, 1991).

The use of reliable and well-validated HRQoL measures in patients with anaemia allows assessment of the impact of the condition and, importantly, assessment of the efficacy of treatment. However, many of the published QoL studies in anaemic cancer patients have tended to employ generic and non-specific scales such as the WHO Performance Status Indicator (Ludwig *et al*, 1993; Mittelman *et al*, 1997) and the Linear Analogue Scale Assessment (LASA, also known as CLAS, Cancer Linear Analogue Scale) (Abels, 1992, 1993; Case *et al*, 1993; Vijayakumar *et al*, 1993; Glaspy *et al*, 1997).

The Functional Assessment of Cancer Therapy-Anaemia (FACT-An) is an anaemia specific scale which was developed in the late 1990s specifically for use in anaemic cancer patients (Cella, 1997a,b). It is a condition-specific quality-of-life instrument, developed to assess the impact of fatigue (FACT-F) and other anaemia-related symptoms on quality-of-life in patients with cancer, which forms part of the FACIT (Functional Assessment of Chronic Illness Therapy) quality-of-life measure. The psychometric properties of the anaemia (FACT-An) and fatigue (FACT-F) subscales with cancer patients have been assessed (Yellen *et al*, 1997), and the FACT-An has been validated as an outcome measure for cancer related anaemia (Cella and Bonomi, 1996; Cella, 1997, 1998; Cella and Webster, 1997; Yellen *et al*, 1997).

The relationship between haemoglobin level and quality-of-life in anaemic cancer patients has been assessed using both the FACT and other measurement scales. Studies using the FACT have found that both the anaemia specific (FACT-An) and fatigue-specific (FACT-F) scales significantly differentiate patients by haemoglobin level (Yellen *et al*, 1997; Cella, 1998). Cella and Webster (1997) used the FACT-An to compare the quality-of-life of patients whose haemoglobin levels were ⩽12 g dl^−1^ with those having haemoglobin levels >12 g dl^−1^. The FACT-An was able to discriminate between patients according to haemoglobin levels; those with haemoglobin levels >12 g dl^−1^ reporting significantly less fatigue (*P*=0.01), fewer non-fatigue anaemia symptoms (*P*=0.016), better physical well-being (*P*=0.003), better functional well-being (*P*=0.001), and higher total (FACT-G) quality-of-life (*P*=0.003) than those with lower haemoglobin levels. Studies using measurement scales other than the FACT have generally found positive associations (moderate but statistically significant) between haemoglobin level and quality-of-life score (Abels, 1993; Case *et al*, 1993; Leitgeb *et al*, 1994; Ludwig *et al*, 1995; Glaspy *et al*, 1997; Malik *et al*, 1998; Sweeney *et al*, 1998).

While associations have been found between haemoglobin levels and quality-of-life in cross-sectional analyses, follow-up analyses of treated patients have also generally shown that the greater the increase in haemoglobin levels following Epoetin alfa (recombinant human erythropoietin) treatment the greater the improvement in quality-of-life as assessed by the FACT (Glaspy *et al*, 1997; Demetri *et al*, 1998; Littlewood *et al*, 1998; Cleeland *et al*, 1999). Indeed, a review of the literature by Beguin (1998) concluded that Epoetin alfa therapy is associated with substantial improvements in quality-of-life and, moreover, that the improvements are proportional to changes in haemoglobin level. The influence of cancer type on response to Epoetin alfa treatment has been explored in various studies (Abels *et al*, 1991; Ludwig *et al*, 1993; Glaspy *et al*, 1997; Demetri *et al*, 1998), and reviewed by Adamson and Ludwig (1999). Some studies show no effect of cancer type (Abels *et al*, 1991; Glaspy *et al*, 1997; Demetri *et al*, 1998) and some report a differential effect (Abels, 1993; Ludwig *et al*, 1993; Adamson and Ludwig, 1999).

The aim of the present study was to investigate further the relationship between quality-of-life and haemoglobin levels within the population of anaemic cancer patients diagnosed with one of four cancers. Other studies have tended to involve patients with a fairly broad range of tumour sites and as a consequence it is difficult to be certain of the relevance of the results to individuals of a particular cancer type. As a measure of HRQoL, two multidimensional measures, the FACT and the SF-36, were utilised. The SF-36 is a generic quality of life questionnaire measuring eight multi-item dimensions (Ware and Sherborne, 1992) which has been widely validated with varied patient groups (McHorney *et al*, 1994). While we expect to find similar associations between haemoglobin and quality-of-life in terms of scores on both the generic SF-36 and the condition-specific FACT, we expect the FACT, and in particular its FACT-An and FACT-F scales, to be more strongly associated with haemoglobin levels.

## PATIENTS AND METHODS

### Patients

On the basis of data from previous studies, sample size calculations suggested that a sample size of 400 patients would be required to detect clinically significant differences in FACT-An score between haemoglobin groups. It was estimated that this sample size would be reached by recruiting all cancer patients diagnosed with one of the four cancers (lung, ovarian, breast or multiple myeloma) who had appointments in six clinics across the UK between the beginning of May 1999 and the end of October 1999. These patients were sent an introductory letter that provided details of the study and informing them that at their next clinic visit they may be asked to participate in the study. At this visit their haemoglobin levels were assessed, and if there was adequate clinic time, those patients with anaemia were asked if they would like to participate in the study by completing the questionnaires. Anaemia was defined as haemoglobin ⩽12 g dl^−1^ in females and haemoglobin ⩽13 g dl^−1^ in males (Sweeney *et al*, 1998). All patients selected were to have a life expectancy of greater than 3 months. Patients receiving all active therapies were included (chemotherapy and radiotherapy). Patients on high dose chemotherapy regimes were excluded.

### Study design

This was a cross-sectional, multi-centre study recruiting patients from six sites across the UK. The study protocol was approved by the Multi-Centre Research Ethics Committee West Midlands Region and ratified by local Ethics Committee. Patients were initially contacted by post, with a consent form being signed when the patient arrived at the clinic. A clinical data sheet was completed for each individual, which included all relevant clinical data (e.g. haemoglobin level assessed as part of routine assessment, primary and secondary cancer sites, time elapsed since diagnosis etc.). The quality-of-life data was collected on the same day by patient completion of two separate instruments: the FACT and the SF-36.

### Data analysis

The FACIT measure includes one General scale (the FACT-G: Functional Assessment of Cancer Therapy – General) composed of 27 general questions divided into four primary quality-of-life domains (physical well-being, social/family well being, emotional well being and functional well being), plus a 20-item anaemia specific scale – ‘Anaemia’. This specific scale comprises 13 fatigue related items (‘Fatigue’) and a further seven non-fatigue anaemia specific items (‘Non Fatigue’). The resulting FACT-F score is the sum of the FACT-G and the ‘Fatigue’ items, while the FACT-An is the sum of all the items – the FACT-G and the ‘Anaemia’ items. Item scores on the FACT were coded and summed for each dimension, according to directions in the FACIT Manual (Cella, 1997a). Each question in the measure is scored, either directly or after score reversal, from 0 (worst possible QoL) to 4 (best possible QoL). Resulting scores range from 0 (poor QoL) to the maximum score (best QoL). The maximum score available differs from scale to scale, depending on the number of items in the scale (see Table 2

**Table 2:**
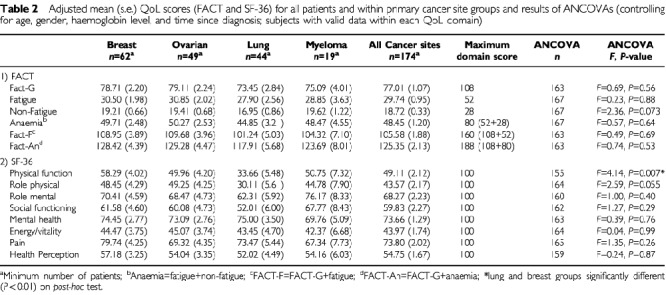
Adjusted mean (s.e.) QoL scores (FACT and SF-36) for all patients and within primary cancer site groups and results of ANCOVAs (controlling for age, gender, haemoglobin level, and time since diagnosis; subjects with valid data within each QoL domain)

). For each SF-36 dimension (Physical Functioning, Role Limitations due to Physical Problems, Role Limitations due to Emotional Problems, Social Functioning, Mental Health, Energy/Vitality, Pain, and General Health Perception) item scores were coded, summed and transformed onto a scale from 0 (worst possible health state) to 100 (best possible health state). Data are summarised by means of frequency counts and descriptive statistics. Missing data in the FACT were treated as the FACIT group recommends, by prorating the scale score when less than 50% of the items in that scale were missing (Cella, 1997a). The developers of the SF-36 recommend the use of an algorithm for substituting missing data, however Layte and Jenkinson (1997) do not recommend this approach for the UK SF-36. The SF-36 missing data were not imputed in this study.

Linear regression analyses (with haemoglobin, which here has a linear relation with QoL score, used as a continuous variable) were conducted to investigate the extent to which haemoglobin and other variables such as age, gender, and time since diagnosis contribute to QoL (SF-36 domains and the FACT subscales). Partial correlations (correlations with the values of other potentially confounding variables held constant) were then conducted between haemoglobin level and the FACT-An and FACT-F scales and the SF-36 domains to investigate further the relationship between haemoglobin level and QoL. The partial correlations were conducted controlling for the confounding variables of age, gender and time since diagnosis and on the patients with valid data on all QoL domains. Analyses of covariance (ANCOVA) were used to test for statistical significance in the QoL scores among the four cancer groups, controlling for the same covariates (age, gender, time since diagnosis) plus haemoglobin level.

In order to visualise the relationship between haemoglobin level and QoL, patients were subdivided into four haemoglobin groups of as equal size as possible at the haemoglobin quartiles: lowest through 9.8, 9.9 to 10.9, 11.0 to 11.5, and 11.6 through highest. This maximises the statistical power for comparisons among groups. Mean quality-of-life scores were compared among the groups using analysis of variance (ANOVA) and analysis of covariance (ANCOVA), controlling for the same covariates as in the partial correlation analysis. *Post-hoc* testing was used to make individual comparisons among groups. The significance of any linear trend was assessed within ANOVA by dividing the heterogeneity among the groups into that due to linear trend and that due to departures from linear trend. All analyses were carried out using SPSS version 9.0 for Windows. The significance level for all analyse except the *post-hoc* tests (for which the *P*-value was set at *P*<0.01 to control for multiple comparisons) was set at 2-sided *P*<0.05. However, on account of the large number of analyses performed, those associations significant at the 1% level (*P*<0.01) are considered most reliable.

## RESULTS

### Description of sample

On account of low patient recruitment, the recruitment period was extended for 6 months to the end of May 2000. It was not practicable to extend the recruitment period further. One hundred and seventy-nine anaemic cancer patients completed the study questionnaires. Across the six study centres, between 90 and 100% of those approached agreed to participate. Seventy-three per cent (*n*=130) were female and 27% (*n*=47) male. The mean (s.d.) age of responders was 59 (10) years (min 38, max 84 years). Ninety per cent of patients (*n*=159) were currently receiving some form of treatment, with the majority of them 148 (90%) receiving chemotherapy compared with seven (4%) receiving radiotherapy and four (3%) mixed chemotherapy and radiotherapy regimens. The mean (s.d.) time elapsed since diagnosis was 18 (31) months (min 1, max 192 months) and the majority of people (89%, *n*=137) were outpatients.

**Figure 1:**
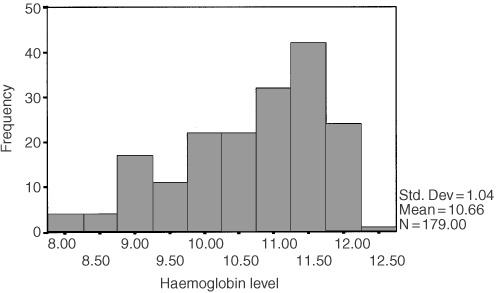
Frequency distribution of haemoglobin levels.

In terms of clinical diagnosis, 36% of patients (*n*=64) had a diagnosis of breast cancer, 28% (*n*=51) ovarian cancer, 25% (*n*=45) lung cancer and 11% (*n*=19) multiple myeloma. Thirty-nine per cent of patients (*n*=70) had secondary cancer sites. The mean (s.d.) haemoglobin level within the sample was 10.66 (1.04) g dl^−1^. The spread and frequency of values is shown in Figure 1

. The haemoglobin distribution was such that, in terms of the quartiles, 25% of the sample had a level of 9.8 g dl^−1^ or lower, 26% a level of between 9.9 and 10.9, 25% between 11.0 and 11.5, and 24% 11.6 or greater. The haemoglobin level did not differ significantly between males and females (mean s.d.) 10.52 (1.08) *vs* 10.70 (1.03) respectively, *t*=1.05, df=175, *P*=0.30).

Table 1

**Table 1:**
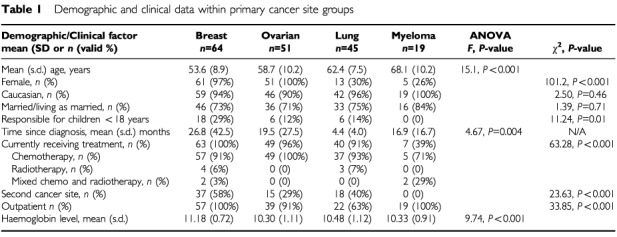
Demographic and clinical data within primary cancer site groups

shows the distribution of demographic and clinical data within primary cancer site groups. The groups differed significantly in terms of a number of factors. As would be expected, the proportion of female patients was highest among those with breast and ovarian cancer. In terms of the difference in age, there was a significant linear trend in age from those with breast cancer, who were the youngest group, through to those with multiple myeloma, who were the oldest (*F*=45.1, df=1, *P*<0.001). Breast cancer patients were also more likely to be currently receiving treatment, to have been diagnosed for a longer period of time (more than two years on average), and to have a secondary cancer site. Patients with lung cancer were more likely to be an inpatient than an outpatient. Haemoglobin levels also differed among the groups, with *post-hoc* tests showing that breast cancer patients had significantly higher haemoglobin levels than all other groups.

### Quality of life scores

After prorating the FACT scores, there was missing data for between one and five patients, and on the SF-36 domains (which were not prorated) for between 3 and 16 patients. Table 2 shows the mean FACT and SF-36 scores for the study patients. Scores are shown overall and individually by cancer site, adjusted for the potentially confounding effects of age, gender, haemoglobin level, and time since diagnosis. While patients with lung cancer tended to report the worst quality-of-life, on analysis of covariance (adjusting for age, gender, haemoglobin level, and time since diagnosis) most quality-of-life domain scores did not differ significantly among the cancer site groups. There was significant heterogeneity among the groups only in terms of the SF-36 Physical function domain scores (*P*=0.007). *Post-hoc* testing confirmed that individuals with lung cancer reported significantly poorer scores on these domains than those suffering from breast cancer. Additionally, and although the associations did not reach statistical significance at the 1% level, patients with lung cancer tended to report poorer SF-36 Physical Functioning than patients with either ovarian cancer or multiple myeloma (*P*<0.05), poorer SF-36 Role Physical scores than patients with either breast and ovarian cancer (*P*<0.05), and poorer FACT non-fatigue scores than patients with all other cancers (*P*<0.10).

### The association between haemoglobin and quality-of-life

#### Correlations between haemoglobin and quality-of-life

Partial correlations (controlling for age, gender, and time since diagnosis) between haemoglobin levels and quality-of-life are presented in Table 3

**Table 3:**
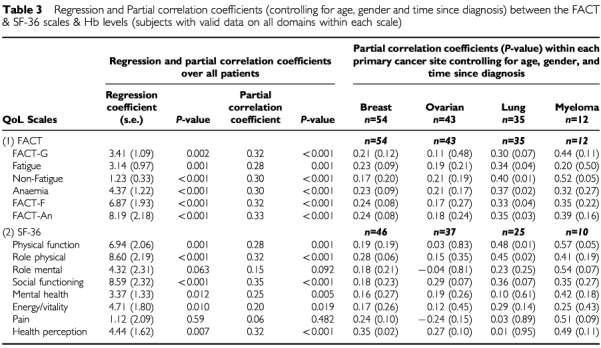
Regression and Partial correlation coefficients (controlling for age, gender and time since diagnosis) between the FACT & SF-36 scales & Hb levels (subjects with valid data on all domains within each scale)

. Significant positive correlations were found between haemoglobin and all the FACT scales (*P*<0.001), indicating that the higher the haemoglobin level the better the quality-of-life. In addition, positive correlations were found between haemoglobin and all of the FACT-G subscales (data not shown). While similar correlations were found between haemoglobin and the SF-36 domains in that the higher the haemoglobin the better the quality-of-life, the associations were weaker in some of the domains (Mental Health and Energy/Vitality) and non-significant in two (Role Mental and Pain).

Positive correlations were observed between haemoglobin and quality-of-life within all four cancer groups (Table 3). The partial correlations for subjects with ovarian and breast cancer were of a smaller magnitude, however, than observed in the overall sample and did not reach statistical significance. Patients with lung cancer and multiple myeloma showed the strongest associations between haemoglobin and quality-of-life, with lung cancer patients showing moderate to strong positive partial correlations between haemoglobin and quality-of-life, with all but one of the FACT scales reaching statistical significance. Sample sizes within the multiple myeloma group were too small for the estimates of statistical significance to be reliable.

#### Regression analyses between haemoglobin and quality-of-life

Multiple regression analyses were conducted to examine the association between haemoglobin and SF-36 and FACT scores adjusting for the effects of age, gender, and time since diagnosis. The results of these analyses in terms of the regression coefficients associated with haemoglobin level are given in Table 3. Haemoglobin level was significantly and independently associated with most FACT and SF-36 QoL domain scores. The regression coefficients were positive indicating that each unit rise in haemoglobin level was associated with an increase in QoL.

Table 4

**Table 4:**
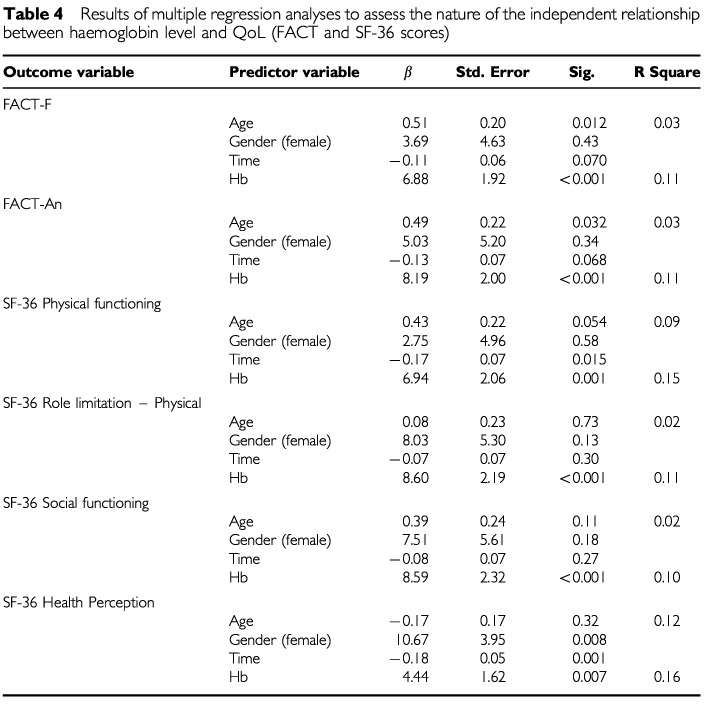
Results of multiple regression analyses to assess the nature of the independent relationship between haemoglobin level and QoL (FACT and SF-36 scores)

shows the full regression results for the regressions on the FACT-F and Fact-An domains, and on the SF-36 Physical Functioning, Physical Role Limitation, Social Functioning, and Health Perception domains. Age, gender, and time since diagnosis were entered first in to the regression model. In terms of the relationship with FACT-F and FACT-An, they accounted for 3% of variability in quality-of-life. Haemoglobin accounted for an additional 8% of variability. The values of the regression coefficients indicate that both haemoglobin and age are significantly associated with quality-of-life, with a unit rise in haemoglobin equalling an average rise of 6.88 units in FACT-F (out of a total score of 160) and an average rise of 8.19 units in FACT-An (out of a total score of 182). Haemoglobin displays a similar predictive pattern for the SF-36 Physical Functioning, Role Limitation-Physical and Social Functioning scales (Table 4). On average, haemoglobin accounted for an additional 7% of the variability of these SF-36 scales with a unit rise in haemoglobin equalling an average rise of 8.00 units (out of a total score of 100). Haemoglobin is slightly less predictive of Energy/Vitality and Health Perception, providing only an additional 2–3% variance over the other variables (age, gender and time since diagnosis).

#### Difference in quality-of-life among haemoglobin groups

In order to visualise the relationship between haemoglobin level and QoL, Table 5

**Table 5:**
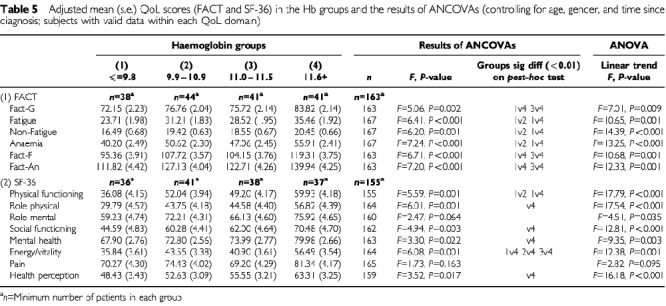
Adjusted mean (s.e.) QoL scores (FACT and SF-36) in the Hb groups and the results of ANCOVAs (controlling for age, gender, and time since diagnosis; subjects with valid data within each QoL domain)

shows the mean quality-of-life scores in the four haemoglobin groups (divided at the quartiles). There was significant overall heterogeneity among the groups in all the FACT subscales and all of the SF-36 domains with the exception of Pain. In general, and as also shown by the correlation analyses reported above, groups with higher haemoglobin levels had better quality-of-life although the differences were most evident between those in the highest haemoglobin level group and those in the lowest group; the subjects in the two intermediate groups reported similar quality-of-life. This may be a reflection of the fact that group 3 has a fairly narrow range of haemoglobin scores. Indeed, in terms of the FACT scale, there were significant linear trends (with no significant departures from linear trend) for increasing quality-of-life with increasing haemoglobin level for each scale. *Post-hoc* testing showed specific differences between the lowest and highest haemoglobin groupings (groups 1 and 4), between lowest and second lowest groups (1 and 2), and between the third and highest quartiles (groups 3 and 4). In terms of the SF-36 domains, significant linear trends were found for all domains with the exception of Pain, with specific differences being found mainly between the lowest and highest groups.

## DISCUSSION

This study provides further support for the positive relationship observed in previous studies between haemoglobin level and quality-of-life in anaemic cancer patients. While the study has a number of limitations, its strengths include the use of both a generic and a specific quality-of-life instrument; the restriction of study patients to those suffering from one of four cancers; the inclusion of patients from multiple centres across the UK; and the ability to control for potential confounding factors to include age, gender, and time since diagnosis. Weaknesses of the study include potential selection bias; any effect of missing data; and the fact that data on various important confounding factors were not collected, such as information on disease response or progression, duration and type of chemotherapy, tumour type, nausea and pain. These factors will clearly influence both haemoglobin levels and quality-of-life, and thus the inability to adjust for their effects is a notable study shortcoming. In addition, the cross-sectional nature of the study means that the association between changes in haemoglobin and changes in quality-of-life cannot be assessed.

One hundred and seventy-nine patients completed quality-of-life assessment forms at the same time as their haemoglobin levels were assessed. This figure of 179 is not quite half of the required number from sample size calculations, a consequence of the low rate of patient recruitment. However, with the exception of the large studies reported by Glaspy *et al* (1997) and Demetri *et al* (1998) the study is still relatively large in comparison with similar studies. Moreover, statistically significant associations were observed in this study between QoL score and haemoglobin level, demonstrating that the study has sufficient statistical power. Thus, in terms of the specific quality-of-life instrument, the FACT (Cella, 1997a,b), the higher the haemoglobin level the fewer the anaemic specific limitations, and the less fatigue, reported. Similarly, for the general items, the higher the haemoglobin level the better the reported quality-of-life. Higher levels of haemoglobin were thus associated with better physical, emotional and functional well-being. As was also observed by Yellen *et al* (1997), who found that it was the lowest (<11 g dl^−1^) and highest (>13 g dl^−1^) haemoglobin groups who differed most, it was the lowest (<9.9 g dl^−1^) and the highest (>11.5 g dl^−1^) haemoglobin groups that in this study reported the greatest between-group difference in quality-of-life score; subjects with intermediate haemoglobin levels generally reported similar quality-of-life. Statistically significant linear trends for increasing quality-of-life with increasing haemoglobin were, however, found for all FACT scales (*P* at least<0.01). In terms of the type of patient group, exploratory analyses found that those with lung or breast cancer contributed most to the correlations seen between haemoglobin and quality-of-life; when analysed separately these patients reported much stronger relationships between haemoglobin and quality-of-life than those with ovarian cancer or myeloma.

In terms of the generic SF-36, similar trends were observed: higher haemoglobin scores were associated with better Physical Functioning, Social Functioning, Mental Health, Energy/Vitality and better Health Perception. All associations but those with Role Limitation and Pain were statistically significant. While there was a significant correlation between the Energy/Vitality domain of the SF-36 and haemoglobin level, the size of the correlation was smaller than expected given the higher correlations between the Fatigue specific domains on the FACT (FACT-F). This is in line with our hypothesis that the generic SF-36 will be less sensitive to anaemia-specific issues than the condition-specific FACT-An and FACT-F. The SF-36 Energy/Vitality domain comprises just four items (‘Did you feel full of life’, ‘Did you have a lot of energy’, ‘Did you feel worn out’, ‘ Did you feel tired’), compared with the FACT-F scale comprising 13 items relating to fatigue related aspects of anaemia. Again, exploratory analyses found that those with lung or breast cancer contributed most to the correlations seen.

In conclusion, this study has utilised a well-validated quality-of-life measure, developed specifically for use with anaemic cancer patients, and concentrated on four types of primary cancer to provide reliable information on the nature of the association between haemoglobin level and quality-of-life in anaemic cancer patients. The nature of the observed associations may be of use in the evaluation of the effectiveness of specific treatments to improve the quality-of-life of anaemic cancer patients. In particular, while no information on haemoglobin and quality-of-life change has been collected in this study, the data are consistent with such observations and suggest that the raising of haemoglobin in anaemic cancer patients would improve their quality-of-life. In terms of the psychometric properties of the condition-specific FACT, the instrument has been shown to provide a more sensitive measure than the generic SF-36 to quality-of-life issues in these anaemic cancer patients and may thus be used alone to assess quality-of-life in this patient group.
